# The impact of the COVID-19 pandemic on individuals with eating disorders: the role of emotion regulation and exploration of online treatment experiences

**DOI:** 10.1186/s40337-020-00362-9

**Published:** 2021-01-12

**Authors:** L. Vuillier, L. May, M. Greville-Harris, R. Surman, R. L. Moseley

**Affiliations:** 1 Department of Psychology, Bournemouth University, Poole, UK; 2 Dorset Healthcare University NHS Foundation Trust, Poole, UK

**Keywords:** Eating disorders, Covid-19, Pandemic, Lockdown, Coping, Emotion regulation, Online therapy

## Abstract

**Objective:**

The Covid-19 pandemic has wrought disruption to everyday life and services, and emerging evidence suggests that those with eating disorders (EDs) are likely to experience marked distress and exacerbation of their symptoms. However, little is known around the most relevant factors to symptom change; whether certain emotion regulation and coping strategies are linked to better outcomes; and how people with EDs are adjusting to psychological interventions moving online.

**Method:**

In a mixed-method design, we collected qualitative and quantitative data from 207 (76 males) self-selected UK residents with self-reported ED, who described and ranked impacts of the pandemic on their symptoms. Regression analysis examined whether emotion regulation strategies were associated with self-reported symptom change, ED symptomatology, and negative emotional states. Thematic analysis explored participants’ experiences of the pandemic, particularly factors affecting their ED, coping strategies used, and experiences of psychological intervention.

**Results:**

Most participants (83.1%) reported worsening of ED symptomatology, though factors affecting symptom change differed between specific EDs. Emotion regulation, such as having fewer strategies, poorer emotional clarity, and non-acceptance of emotions, explained nearly half of the variance in emotional distress during the pandemic. Qualitative findings indicated that difficult emotions (such as fear and uncertainty), changes to routine, and unhelpful social messages were triggering for participants during the pandemic. While some participants described employing positive coping strategies (such as limiting social media exposure), many reported using ED behaviours (among other maladaptive strategies, like alcohol use) to cope with the pandemic. Finally, loss of treatment support, feeling underserving of support and experiencing a ‘detached connection’ online were further exacerbating factors for these participants.

**Discussion:**

While our sample was self-selected and may not represent all people with EDs, our results suggest that people with EDs have been strongly affected by the pandemic. Some aspects of online treatment were found to be beneficial but our findings suggest it also needs some improvement. Our paper discusses implications for online treatment such as taking into account personal circumstances and, in a time where people have limited control over the antecedents of negative emotion, the need to develop skills to manage emotions when they arise.

**Supplementary Information:**

The online version contains supplementary material available at 10.1186/s40337-020-00362-9.

## Plain English summary

The Covid-19 pandemic has damaged mental health on a global scale, and emerging research suggests that people with pre-existing mental illnesses, including eating disorders (EDs), may suffer the greatest deterioration. Aiming to inform future treatment and support, the present study examined which aspects of the pandemic have been especially difficult for people with EDs, and whether certain coping strategies and ways of dealing with emotions have been associated with better wellbeing. We also explored experiences of the move to online psychological treatment. Of 207 participants surveyed online, 83.1% reported that their ED symptoms had worsened, most notably due to difficulties managing emotions like anxiety around the unknown situation, changes in routine and in physical activity. Poorer mental health was associated with having fewer emotion regulation strategies, poorer emotional clarity, and non-acceptance of emotions. Participants outlined some helpful coping strategies during this time (such as limiting social media use) as well as some more detrimental strategies (such as increased ED behaviours and alcohol use). Finally, while some aspects were found to be beneficial, our participants reported a loss of support, feeling underserving of help, and a detached connection with their therapist as barriers to a successful online treatment. Our paper discusses the clinical implications of these findings.

With the first outbreaks reported in late 2019, Covid-19 became a global pandemic with alarming speed. Many governments imposed lock-down and social distancing measures, such as those that started on 23rd March 2020 in the UK. While pandemics and periods of mass quarantine are historically linked with psychological distress and psychiatric morbidity [1–3], their impact is disproportionately felt by people living with pre-existent mental illnesses [4, 5]. Psychological services have valiantly rallied to mobilise online delivery of services [6], but the efficacy of such services and their reception with patients is thus far unknown.

One group of individuals of particular concern are those with eating disorders (EDs), long recognised as among the deadliest psychiatric illnesses [7, 8]. Recent efforts to examine the effects of the pandemic on people with eating disorders suggest a marked worsening in symptoms, most notably in restricting, binge eating, purging and pathological exercising [9–13]. Qualitative exploration of these experiences revealed that individuals with eating disorders attribute the exacerbation of their symptoms to disruption across many spheres of their lives, alongside reduced access to healthcare services [11, 14]. Changes in living situations, for instance, have meant that some people have had to move back in with their parents, which for some may mean more support towards their eating disorder, but for others more anxieties around feeling trapped, watched, or over having less influence over meal choices [11, 14]. Relatedly, being housebound during lockdown has affected access to social support networks, with individuals unable to see friends for support, and incurred changes to exercise routines, with some individuals distressed by the loss of access to gyms and others feeling compelled to exercise more due to the constant availability of online exercise classes and social pressures around it [11, 14]. Participants in these studies also highlighted social pressure in the form of triggering messages on social media, having increased their media consumption. Indeed, people during the lockdown have spent more time engaging with media online and on television [15], and others have raised concerns that greater exposure to home cooking or exercise classes may have negatively impacted individuals with EDs [16]. Finally, the lockdown has resulted in changes in food buying and eating behaviours, with participants reporting food stockpiling and resultant binge eating episodes; justifying food restriction with the reality of food insecurity [17]; or being unable to access ‘safe’ foods. These studies [11, 14] validated many of the concerns raised by clinical researchers [18], who also note the likelihood that the increased anxiety of this period may move individuals from a prodromal to full disease state.

The fact that this is occurring during a time of indefinite disruption to services is deeply worrying. These illnesses frequently run a protracted course [19, 20], with some evidence that this chronicity is underpinned by progressive neurobiological changes which incur treatment-resistance and impede full recovery [21]. It is paramount that changes in symptomatology are mapped and understood in order to inform emerging online paradigms and support individuals in their recovery efforts. In light of this, a valuable area of focus may be emotion regulation and coping strategies. Emotions are known to play a role in the pathology of EDs [22–24], and in this context, it is highly relevant that the pandemic has fuelled additional feelings of depression, anxiety, uncertainty and psychological distress [5, 25]. For example, many people have lost jobs or are facing unemployment, thus raising fears around an uncertain future; others may be experiencing the heightened stress and guilt associated with balancing work and childcare. Many are highly anxious about their physical health and that of their loved ones. One study conducted early in the pandemic (April–May) with a large population of people with EDs found that over 60% of participants were experiencing comorbid generalised anxiety disorder, with participants reporting substantial increase in anxiety, attributed specifically to Covid-19, since the end of 2019 [11]. Other studies reported heightened feelings of sadness, anhedonia, apathy and worthlessness [12, 13]. Identifying and regulating emotions is a known difficulty for people with EDs [23, 26], which means they may be especially poorly equipped to cope with these added difficulties. Indeed, within the same samples, substance abuse, self-injury and suicidal feelings were reported by some participants [11–13]. Some theorists suggest that eating disordered behaviours arise due to maladaptive emotion regulation strategies [23, 26], and the pandemic both increases the likelihood that these maladaptive regulation strategies will be deployed whilst simultaneously barring access to strategies which might have previously helped [18]. As such, the question arises as to which emotion regulation strategies have been used during the pandemic and whether some are associated with less exacerbation of ED behaviours. While studies have suggested that reappraisal and acceptance may be useful strategies during the pandemic [25, 27], as of yet no research has explored the relationships between emotion regulation strategies and psychological wellbeing in people living with an ED during the pandemic. It may however be the case that these emotion regulation strategies explain some of the variance in the impact of the pandemic on individuals with an ED [9, 10, 14], in which case identifying the coping strategies used by people with EDs during the pandemic and which are linked with better outcomes could help develop better treatment plans.

Given that psychological treatment seems likely to continue online for the foreseeable future, another crucial research goal lies in examining how the move to online treatment (e.g. 6) has been received. Disruption to services, routines and accountability have jeopardised recovery efforts for many people with EDs [11, 14], and even impacted the ability of individuals with past eating disorders to remain in remission [11]. In the UK, evidence-based psychological therapies, such as eating disorder focused Cognitive Behaviour Therapy (CBT-ED), are typically offered to adults with eating disorders [28]. The impact of the pandemic on treatment has varied across localities; treatment in some ED services may have been paused or reduced if there was a requirement to redeploy staff into essential areas of the NHS, whereas other services have been able to continue offering treatment by branching into telehealth, for instance video-conferencing. This kind of remote psychological treatment was already effectively employed in areas where geographical distance can make it difficult for individuals to access face-to-face healthcare [29], but does require additional considerations, such as access to adequate internet connectivity, a quiet, confidential space, and in the case of eating disorders, navigating how to conduct in-session weighing [30, 31].

While online treatment for eating disorders may become the norm, little is known at present about the patient experiences and barriers and facilitators of such treatment as deployed in the UK. A very preliminary qualitative analysis of a small group of patients with anorexia nervosa and their families [10] suggested that while some benefited from continued support in this alternative format, others were unable to tolerate seeing themselves on screens. Other concerns expressed by patients and families about online treatment included the lack of non-verbal communication; that previous homework challenges, such as eating out, would become impossible; and that virtual connection placed responsibility on patients to manage their own weigh-ins, an accountability which would be difficult for people at early stages of recovery and which might constitute an extra burden on carers. While research on the COVID-19 pandemic has flourished and there is preliminary evidence of deterioration in people with EDs [9, 10, 14], further in-depth empirical scrutiny is imperative for best supporting these individuals through the present time. The high mortality rates of EDs [7, 8], the development of treatment-resistance over time and the neurobiological changes which maintain chronicity [19–21] all underscore this crucial need for clinical and research attention to people with EDs. Accordingly, the current study had three aims. To inform clinical focus during this period, we firstly aimed to build a more comprehensive account, enabled by a mixed-methods design, of *which* factors were important during the pandemic as well as *how* these factors affected participants’ experiences. As such, we built on previous research by ranking these factors in importance and exploring at a deeper, qualitative level their impacts on participants. With a more extensive and sex-balanced sample than in previous studies, we also aimed to delineate factors that might be impacted by individual differences, such as diagnosis. Second, we aimed to understand whether some emotion regulation strategies were linked to symptom changes and wellbeing outcomes for individuals affected by an ED. We also aimed to further explore this aspect with qualitative reports of the coping strategies people with an ED have used during the pandemic. Third and finally, given that previous studies offered only very sparse consideration of treatment experiences during the pandemic, we qualitatively explored participants’ experiences with online treatment, hoping to inform and optimise future treatment delivery for this vulnerable population.

## Methods

### Participants

Participants self-reporting a current ED diagnosis (*n* = 222) were recruited through a participant recruitment website (Prolific, *n*=168) and through social media (*n*=54). To ensure a degree of homogeneity in treatment options, eligibility criteria required that participants be over age 18 and UK-residents for at least the prior 2 years. Recruiting participants over 18 years of age also allowed us to recruit participants as fast as possible due to the time-sensitive nature of the study. As such, we did not require additional consent from their parent or guardian, and we could use remote recruitments methods that were more suitable for an adult population. Data was excluded from participants who did not clearly specify a singular ED diagnosis (*n* = 3), participants with orthorexia nervosa (*n* = 2), ARFID (n = 2), body dysmorphia or medical issues such as reflux without a DSM-5 classified ED (n = 3), as well as participants who reported a dual diagnosis (e.g. anorexia and bulimia, *n*=4).^1^

The 207 participants included in the study (156 from Prolific) were those diagnosed with AN, BN, BED, or OSFED (or EDNOS if diagnosed before DSM-5) whose demographic details are shown in Table 1. Within the qualitative section, 43 participants responded to questions exploring their experience of treatment during the pandemic. Participants reported ethnicities were white British/Irish/Scottish/European (93.7%), Asian (5.3%), Black (0.5%) and Arab (0.5%).

**Table 1:** Participant demographic information

	*Total*	*AN*	*BN*	*BED*	*OSFED*
Total numbers (males)	*207 (76)*	*91 (29)*	*46 (14)*	*44 (28)*	*26 (5)*
Mean age (SD)	*30.0 (9.7)*	*28.7 (9.1)*	*31.8 (9.2)*	*32.6 (10.7)*	*28.8 (9.7)*
Duration of illness (SD)	*7.3 (7.5)*	*7.9 (8.0)*	*9.1 (7.9)*	*5.0 (5.4)*	*6.1 (6.8)*
In treatment (n)	*42*	*21*	*11*	*7*	*3*
Mean total EDEQ scores (SD)	*4.0 (1.1)*	*4.0 (1.2)*	*4.1 (0.9)*	*4.0 (1.1)*	*4.2 (1.1)*

Males and females were unevenly distributed across the four diagnostic groups (χ2 (3) = 18.8, *p* < .001), with female participants more likely to be found in the AN, BN or OSFED group, and males more likely to be found in the BED group.

There was no difference in duration of illness (t (219) = −.822, *p* = .412), mean age (t (218) = 592, *p* = .555) or symptom severity (t (220) = −.228, *p* = .820) between our samples recruited on Prolific and Social Media. There was however a significant sex difference, as we actively tried to recruit males in our Prolific sample (χ2 (1) = 23.0, *p* < .001).

The diagnosis groups differed significantly in age (F [3, 202] = 3.19, *p =* .025), and in duration of illness (F [3, 202], 2.7, *p =* .047) but were equally matched in EDEQ scores (*p =* .776).

### Materials and procedure

Qualitative and quantitative data are often favourably integrated [32], for instance where researchers hope to comprehend statistical findings at a deeper, more contextual and holistic manner, and validate findings across multiple types of analysis. This motivated our decision to adopt a mixed-methods approach so as to complement quantitative data around the impact of the pandemic with insights around how it affected participants in the greater context of their lives. The impact of the pandemic on mental health and eating symptomatology was quantified with the Depression, Anxiety and Stress Scale [33], the Eating Disorder Examination Questionnaire [34] and a single numerical item in the qualitative survey. Emotion regulation strategies, as predictors of these impacts, were operationalised with the short form version of the Difficulties in Emotion Regulation Scale [35]. Richer details around the impact of the pandemic on ED symptoms, coping strategies and (if relevant) experiences of online treatment, were accrued with a short qualitative survey. The survey took approximately 45 min to complete, including additional questionnaires not reported here, and participants were rewarded with money (Prolific) or a chance to be entered in a prize draw for Amazon vouchers (social media). See supplementary material (item 1) for the detail of the measures and qualitative questions used.

### Data analysis

Our three-part analysis addressed our three research questions. First (Part A), we looked at whether our participants reported a deterioration in ED symptoms due to the pandemic and then, specifically in those whose symptoms deteriorated, which of these factors were the most influential. To examine the differential importance of each variable for participants along the ED spectrum, we conducted one-way ANOVAs (diagnosis as the between-group factor) with Bonferroni-corrected post-hoc tests. To get a clearer picture of our data in this process, we computed non-parametric correlations between these ordinal variables (see supplementary materials, 2); in addition, thematic analysis was carried out on the qualitative responses to understand participants’ experience of these pandemic-related factors in more detail.

Secondly (Part B), we used multiple linear regressions to examine whether some emotion regulation strategies were linked to better outcomes during the pandemic. While predictors were the six DERS subscales, three outcome variables were examined: the total score on the DASS (Model 1), the numerical item within the qualitative survey where participants rated the impact of the pandemic on their eating disorder behaviours (Model 2), and the total score on the EDEQ (Model 3). We used the backward method to remove all non-significant predictors. Again, qualitative descriptions of experiences of coping were analysed as part of our thematic analysis, to identify key themes exploring how participants described coping during the pandemic.

Thirdly (Part C), thematic analysis of participant’s responses about treatment was carried out in order to explore participants’ experiences of coping and therapeutic interventions during the pandemic.

Qualitative data for each section were imported into NVivo 12 (QSR International, 2018) software, retaining the original punctuation, spelling mistakes and grammatical errors. Thematic analysis was then carried out using Braun and Clarke’s (2006) five stage process: 1) familiarisation with the data through initial reading, 2) generating initial codes, 3) collating codes into themes, 4) defining and labelling themes, and 5) selecting illustrative quotes (participant quotes were anonymised, with gender (F or M), participant code, and ED diagnostic label, presented in brackets alongside each quote).

For each part of this analysis, a data trail was kept throughout this process using concept cards. Three researchers analysed the qualitative data (MGH, LM and RS). Initial findings were then discussed within the research group to come up with a holistic set of themes. Second coding was carried out on approximately 14% of the qualitative data and any discrepancies were resolved through discussion.

The thematic analysis findings are described in each of the three sections: Experiences of the pandemic (described in Part A and comprising four main themes), coping with emotions (described in Part B and comprising two main themes), and treatment experiences (described in Part C and comprising two main themes). Figure 2 in the result section outlines all themes together as interrelated factors impacting upon ED symptomatology.

## Results

### Part a: overall impact of the pandemic and factors involved

#### Quantitative analysis

When asked about the overall impact of the pandemic on their ED, the majority of our participants (83.1%) reported that their symptoms had worsened, while 6.8% reported that their symptoms got better and 7.7% reported no change in symptoms (the remaining 2.4% failed to respond to this item). Reported changes in symptomatology did not differ between diagnostic groups (χ2 (18) = 18.8, *p* =.409), although see supplementary materials (3) for a breakdown per diagnoses.

**Fig. 1:**
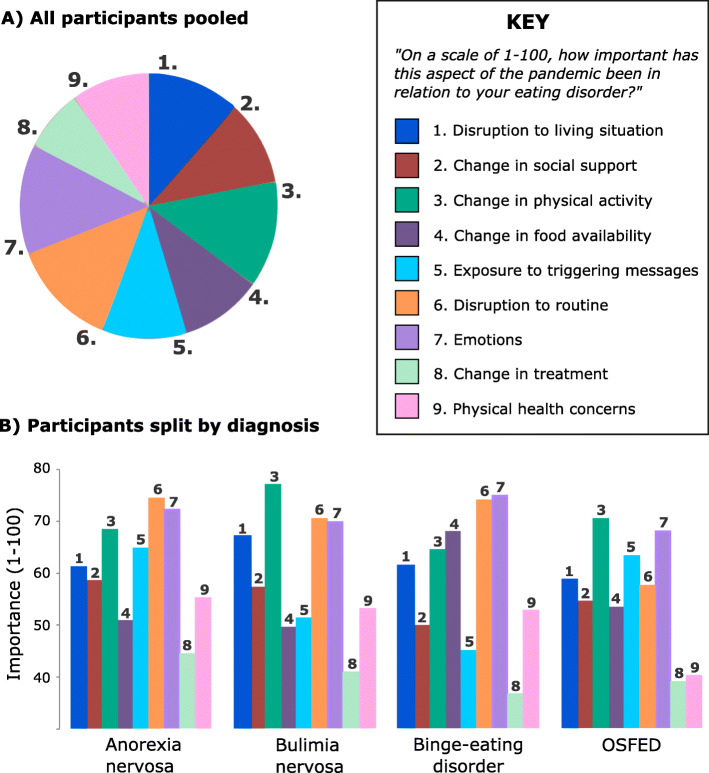
Deleterious impacts of different aspects of the pandemic. Note. In all participants who reported an exacerbation of ED symptomatology, Part **a** depicts the relative importance of 9 different aspects of the pandemic to this worsening of ED symptoms. Part **b** reflects the relative importance of these different aspects when participants were categorised by diagnostic group

Looking specifically at the participants who reported an exacerbation of ED symptomatology (*n* = 172), we examined which factors participants thought had had the greatest impact on their ED. There was striking heterogeneity between participants. Overall, as can be seen in Fig. 1 (and supplementary materials 4), the three most important factors were changes to routine and physical activity, and difficulties around their emotions. Non-parametric correlations between these factors were positive but weak, indicating their independence from one another.

Interestingly, the importance of some factors differed between diagnoses. This was the case for exposure to triggering messages (*F*(3, 171) = 3.4, *p =*.020) which Bonferroni-corrected posthoc tests revealed was more important for people with AN and people with OSFED than for people with BED (mean difference = 19.7, *p =.*005; mean difference = 18.5, *p* = .039, respectively). Differences in the importance of changes to food availability were marginal (*F*(3, 171) = 2.6, *p = .*056) but, in corrected posthoc comparisons, this factor was more important for people with BED than for people with AN (mean difference = 17.1; *p = .*013) or for people with BN (mean difference = 18.4, *p* = .019). Non-parametric correlations between the ‘difficulties with emotions’ factor and the other factors were also illuminative in so far as suggesting which factors were most associated with strong negative emotions. For AN, corroborating the above, exposure to triggering messages was the factor most strongly correlated with emotions for participants with AN (*r* = .506, *p* < .001). The factors most strongly related to negative emotions were physical health concerns in BN (*r* = .637, *p* <.001); change in social support in BED (*r* = .446, *p* = .004); and disruption to routine in OSFED (*r* = .562, *p* = .004).

#### Qualitative analysis relating to overall impact of the pandemic

**Fig. 2:**
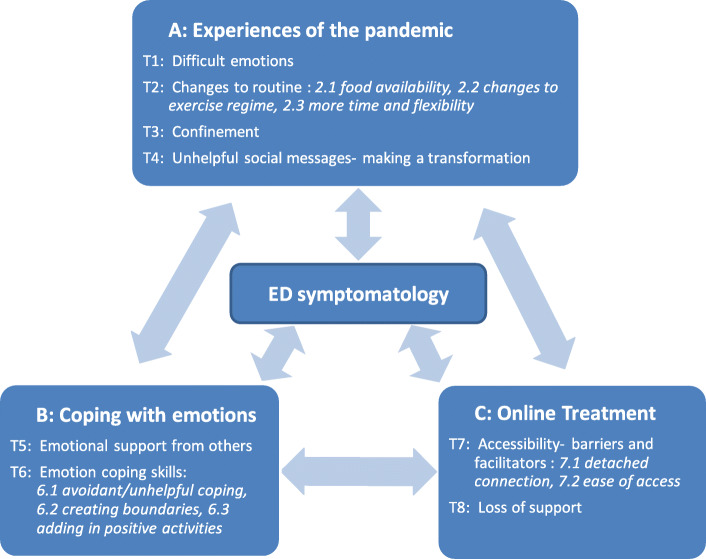
Qualitative themes (T1-T8) and subthemes for experiences of the pandemic, coping with emotions and experiences of online treatment

In free-text responses, we interpreted four main themes around experiences and impact of the pandemic. These are indicated in Fig. 2 (part A, themes 1–4) and described as follows:

##### Theme 1: difficult emotions

Participants reported experiencing a greater level of distressing emotions during the pandemic, which had a negative impact on their ED. This mirrors our quantitative finding that difficulties with emotions were one of the main factors impacting on the deterioration of ED symptoms.

*The pandemic situation has worsened how hopeless and depressed I feel and in turn i think my eating disorder symptoms has become a little [w] orse when my mood is low or things dont seem to be going right I tend to always turn to my eating disorder to cope. [F119, AN]*

Many participants described fear and/or uncertainty during the pandemic exacerbating their ED behaviours. Fear for the health of loved ones, fear of getting the virus, the trauma and distress of having had Covid-19 themselves, or having experienced illness or death of people they knew took an emotional toll. For participants who had been furloughed, or lost their job roles or university routines, the lack of structure and purpose, lack of control and loss of income resulted in distress. Many participants reported feeling a lack of motivation, increased anxiety, and a low mood due to having their work or education taken away from them.

*Lost all my voluntary jobs. Felt like the floor had opened up and swallowed me , felt very very lost and confused*. (*F17, AN):*

ED behaviours were a source of comfort for some, or allowed participants to distract themselves from the situation and their difficult feelings.

*“restriction &b/p [binge-purge] are helpful to me as it distracts my mind from what i am worried about. i only have to focus on food related feelings which is usually easier than the difficult emotions i am avoiding” [F96, AN]*

Some participants described how changes to ED behaviours during lockdown had resulted in changes to their weight during lockdown. Inevitably weight gain added to distress levels and often further exacerbated their ED.

##### Theme 2: changes to routine

Changes to routine during the pandemic resulted in more accessibility to food and exercise, as well as increased time and/or flexibility to engage in ED behaviours. These factors are outlined in the following three subthemes; ‘food availability’, ‘changes to exercise regime’ and ‘more time and flexibility’.

*Food availability.* Initially during lockdown participants described anxiety about food shortages and lack of availability of their preferred or ‘safe’ foods. For some, food shortages resulted in reduced bingeing and purging, or encouraged restrictive eating behaviours.

*At the very beginning due to the lack of food on the shelves at the supermarket , i did feel it was pointless trying to eat as the safe stuff was rarely available. (F17, AN)*

Greater access to food due to stockpiling increased anxiety and perceived lack of control over eating.

*A combination of constantly being at home, with easy access to the kitchen, and doing much larger weekly shops … has caused me a huge struggle with feelings of loss of control over food (F102, BN):*

Food availability at home caused many participants to struggle with regulating their food intake. For participants with more restrictive eating behaviours (such as those with AN), reduced access to ‘safe’ food was more problematic, whereas participants who struggled with binge-purge or overeating (particularly participants with BN or BED), described stockpiling and the increased access to food as home particularly triggering.

*Changes to exercise regime*. In line with the quantitative findings, the limitations around leaving the house and the closure of gyms resulted in reduced opportunities to exercise for many, which was particularly difficult for participants. Lack of routine exercise during lockdown led to worries about reduced energy expenditure and anxiety about how this would affect their weight and shape. This was triggering, resulting in distress, and in some cases, increased compensatory strategies.

*I gained weight, had increased anxiety and felt out of control. My weight had been stable for the past 3 years and I gaining weight in lockdown and not having access to the gym and seeing my body change was a trigger. (F16, EDNOS)*

Changes to the ways in which participants could exercise safely, meant that exercising became a further cause of stress, rather than a way to cope. Some participants substantially increased their exercise regime at home due to more free time, or to deal with their lack of control in other areas of their lives.

*More time and flexibility*. As a result of the restrictions put in place during the pandemic, participants described significant reductions in their routine activities, social events or work schedule. More free time meant increased boredom and lack of distraction, which resulted in greater focus on negative thoughts, eating and weight.

*Being inside because of covid also leaves me with so much time to spare and think about being hungry and then eating and then resenting myself and my body. There is too much time to think and nothing to distract me from obsessing about food (F115, BN)*

Working from home allowed some participants more freedom and flexibility to engage in ED behaviours. For example, by exercising during lunch, or engaging in more restriction, bingeing or purging.

*Because I am working from home, I am able to go for runs before work and on my lunch break which is great. (F127, AN)*

Lack of value, purpose and routine resulted in lack of motivation for many. This hindered engagement in ED recovery.

*In the initial stages of the pandemic it seemed pointless to try to manage my ED. The stress and anxiety regarding the future meant that I did not see the point in investing energy in my own physical or mental health … (F88, BED):*

However a few participants felt that the free time was valuable for recovery, for instance they were able to focus more on healthy eating behaviours and implementing coping strategies.

##### Theme 3: confinement

Participants who were living alone described feeling confined and isolated. The lack of social connection led participants to engage in more ED behaviours with no one there to monitor them or be accountable to.

*It has meant that i have no one around to keep an eye on me so i can binge when i want to and then make myself sick to make myself feel better and i don't have to explain it to anyone because there is no one there to hear me … (M44, BN)*

Although confined isolation was helpful in coping with the threat of the virus, participants who usually used activities and social interaction as ways to cope with their ED were left feeling trapped and less able to cope.

*A usual coping mechanism before was to prevent periods of isolation when I thought Ed might surface. Haven't been able to use that strategy. [F13, AN binge purge subtype]*

In contrast many participants moved home, or spent lockdown with family, partners or friends. Participants described stress at the lack of privacy, and feeling constantly monitored by others. This resulted in distress, low mood, conflict or withdrawal.

*… I am finding it increasingly harder to constrict as my family are able to monitor what I am eating and my every move. I have screaming matches every day with my parents which isn't good for anyone, especially my sister so I am feeling more depressed than usual. (F89, AN):*

Sometimes living with others also resulted in less control over what participants could eat, or pressure to eat certain foods, causing greater distress. These living arrangements resulted in more secrecy and attempts to hide behaviours.

*It's meant I've had to go out of my way to hide food. For example a whole cooked chicken in my rucksack (M39, BED):*

While the support from families and friends was valued by many, some participants described the stress of family life as a trigger for their ED. Participants who were caring for others, looking after their children or working from home, described the stress and exhaustion, with no time or space to focus on caring for themselves.

*Crazy me and husband working at home for first time, with two teenagers who are not doing school work, out of control of situation in my own home as I have to sit at a desk at home 8 hrs a day (F118, BED)*

However, some participants reported that confinement meant that they were facing fewer stress provoking situations due to lack of work and social pressure. This had reduced some of their daily stressors.

##### Theme 4: unhelpful social messages- making a transformation

The pandemic was promoted online as a time to make a transformation, lose weight and get fit, with access to more online content promoting work outs and diets. These messages were unhelpful in promoting ED behaviours, or increasing pressure to conform to these ideals.

*Everywhere I look there are people doing workouts or eating healthily or talking about weight gain/loss during lockdown. Theres a pressure to come out of this being amazing and self sufficient and healthy and people are promoting it. (F24, BN)*

Some participants found that their increased free time during lockdown led to them making more social comparisons on social media, which triggered their ED and negative thoughts.

*I have spent more time on my phone and using instagram, this is harmful as i compare myself to others and this makes me feel bad about myself. [F126, AN]*

Messages about fear of gaining weight during lockdown were also triggering, exacerbating these fears in participants at a time when gyms and routine exercise were less accessible. In line with quantitative findings, triggering social media messages were mostly described by participants diagnosed with AN.

*I have been triggered a few times from people posting things like Don't worry about gaining weight in quarantine/other body positive messages, because prior to seeing them I didn't even think that weight gain could be a potential issue of quarantine. (F15, AN)*

However, a small number of participants described how these types of messages did not affect them, as their ED was more fuelled internally.

### **Part B: the role of emotion regulation**

#### ***Quantitative analysis***

All assumptions for multiple regressions were met. Differences between diagnostic categories were slight, so for clarity we only present the regression analyses for the whole sample. The results of regression analyses split by diagnosis categories can be found in supplementary materials 5.

##### Model 1: DASS

We found that reduced access to emotion regulation strategies (B = 3.33, *p <.*001), non-acceptance of emotions (B = 1.57, *p = .*002), and reduced emotional clarity (B = 1.50, *p = .*001) were significant predictors of poor mental health as measured by the DASS (*Adj. R*^*2*^ = .472, *F*(3, 203) = 62.3, *p <*.001).

##### Model 2: self-reported change in symptoms

We found that difficulties maintaining goals when upset (B = 0.09, *p = .*004) was the only significant predictor of self-reported changes in eating disorder symptoms (*Adj. R*^*2*^ = .036, *F*(1, 200) = 8.54, *p =* .004).

##### Model 3: EDEQ

We found that non-acceptance of emotions (B=0.07, *p=.*007) and difficulties maintaining goals when upset (B = 0.09, *p =.*001) were significant predictors of eating psychopathology (*Adj. R*^*2*^ = .156, *F*(2, 204) = 20.0, *p <*.001).

#### ***Qualitative experiences relating to coping with emotions***

We interpreted two themes relating to coping with emotions; Emotional Support, and Emotion Coping Skills (See Fig. 2, part B, themes 5 and 6). With reduced access to usual emotion regulation strategies, such as emotional support from loved ones, participants spoke about the increased use of other coping strategies.

##### Theme 5: emotional support from others

ome participants described how supportive family members had been protective for them during the pandemic. Talking to family, partners, or friends was a key strategy for coping with their eating disorders for many participants*.*

*I am also a lot more honest with my family and friends about the struggles I face so I have constant support from them when I need it. [F122, AN]*

There was a reduced amount of contact with friends or family members during lockdown and many struggled with having less connection and less emotional support from loved ones.

*Not speaking to my best friends every day / not [seeing] them at all has made things worse. [F99, BN]*

The lockdown also meant a loss of support group activities. However, some participants also spoke about finding new avenues for support, with some support groups moving online and this making them easier to access. There was also an increased use of recovery-focused social media use and eating disorder support websites.

*I feel this pandemic has put a lot of services online that are easily accessible for help. I discovered an online zoom support group for people affected with eating disorders and this has helped me every week. I feel I can talk openly and feel less alone. My normal service has not been active during this time. [F7, AN]*

In contrast, a few participants described how they did not rely on support from others and tended to deal with their difficulties on their own. For some, there was a reluctance to talk about their own issues.

*Feel guilty about talking about any difficulties I have during the pandemic as there are people who are in much worse circumstances, and my difficulties are little in comparison. [F26, AN]*

These participants spoke of their difficulties as feeling insignificant in comparison to the pandemic and/or not wanting to worry others at a stressful time.

##### Theme 6: emotion coping skills

Participants discussed different strategies used to cope with emotions, both helpful and unhelpful, as outlined in three subthemes; ‘avoidant or unhelpful coping strategies’, ‘creating boundaries to look after self’, and ‘adding in positive activities’.

*Avoidant or unhelpful coping strategies*. Some participants described drinking alcohol and increasing their use of medication, such as sleeping pills and painkillers, as a short-term escape mechanism.

*I’ve been drinking way more than I should as a means to escape the reality for a short period of time.” [M72, BED]*

A few participants reported an increase in self-harm as a coping strategy; some felt as if this was their only outlet, as their other normal coping mechanisms had been restricted.

*The pandemic has increased my self harm, as I’ve felt so restricted by the government in terms of what I can do to deal with my feelings (i.e. initially – only exercise once a day, not able to exercise with anyone else). Using healthy means to deal with difficult feelings (i.e. go for a walk, meet a friend for coffee) have been more limited and so it is really easy to go back to unhelpful ways of coping such as self harm. [F5, AN]*

Participants who spoke about their increased drinking, self-medicating, and self-harm recognised these as unhelpful coping strategies.

*Creating boundaries to look after self*. There were several coping strategies described which were about taking positive steps to look after participants’ emotional wellbeing. Getting enough sleep was one important factor for helping participants to feel able to cope with their difficulties. Some participants focused on planning appropriate meals to avoid comfort eating or takeaways. Others also put strategies into place to allow themselves to exercise for its positive effects but to use limits avoid over-exercising.

*“I have been exercising more … which, as I said, has me in two minds. More exercise can't be bad for me I'm sure, but I'm worried it will become obsessive, to stop this I've set a strict timer and forced myself to stop when the alarm goes off no matter how I feel, then I immediately go for a shower so I am not tempted to carry on.” [M53, BED]*

Participants also recognised that the news and social media contributed to increased anxiety and so it was important for their exposure to be limited.

*I usually have a good handle on my emotions per se. I had a spike in anxiety in the beginning though. As I mentioned, this seemed to be fuelled by the media. I consequently took my usual all-or-nothing approach (which is often an issue with the eating) however it worked well with regard to abandoning the media and news. This I believe reduced my anxiety by a notable degree. [M61, BED]*

Many participants also coped by limiting their time on social media, or deactivating their social media accounts completely.

*So much bullshit pressure to get a lockdown body - for this reason I came off social media early on in lockdown. [F76, EDNOS]*

Implementing boundaries demonstrates a helpful way to cope with distress as discussed in the earlier theme of ‘unhelpful messages – making a transformation’.

*Adding in positive activities*. Participants recognised that unstructured time could have a negative impact on their emotional wellbeing and their eating disorders. Again, this fits with our quantitative findings in which participants rated changes to routine as having one of the greatest impacts on the worsening of the ED symptoms. Participants found that building structure, developing new routines and planning mood boosting activities were beneficial for their mood and EDs.

*Planning what to do the next day- gives me some routine and something to actually wake up for! [F31, AN]*

There were a wide range of positive activities which helped people to cope. Participants spoke about enjoying reading and learning new skills. Having a form of creative outlet, such as oil painting, photography, was important for some participants to channel their stress and express themselves.

*Trying to keep myself busy, I have taken up crafts as a mean to express myself and take my mind off things. [F116, AN]*

Another popular coping strategy was using different forms of writing, including gratitude diaries and stories.

*My best and favourite it probably noting things down, whenever I come across a problem that requires a lot of thought I open a note page and jot everything … for example, I come across a problem that gives me a lot of anxiety the best way for me to get rid of it would be to understand the situation at hand therefore I write out different notes that give me a better understanding, why its happening, whats next, best course of action for minor benefits and major benefits etc. [M59, AN]*

Participants spoke about the benefits of going outside and enjoying time with their dogs as helpful coping strategies.

*When I feel down and want to engage in unhelpful behaviours I have asked friends to borrow their dog for a few days to help me cope. Dogs are happy 99% of the time, require walking which means I have to get out of bed and face the world, I can hug them (even though I can’t hug humans outside my household which I miss) and they don’t care what size my body is or what it looks like. [F5, AN]*

The range of positive activities discussed were beneficial for people’s wellbeing and participants also found that keeping busy was a helpful distraction.

### Part C: qualitative experiences relating to online treatment

Participants who had received some form of treatment, either by telephone or by online videoconferencing, spoke about their experiences. Participants who did not receive any treatment during the pandemic also shared their experiences on why that was the case. Themes interpreted in this part of the analysis are depicted in Fig. 2, part C, themes 7 and 8. The first theme captures aspects of remote sessions which impacted the accessibility of this support. The second theme identified an experience of loss, including those who had no support from healthcare during the pandemic and those whose experience fell short of services usual provision.

#### Theme 7: accessibility - barriers and facilitators

There were two subthemes reflecting some of the benefits and disadvantages to remote sessions: ‘detached connection’ and ‘ease of access’.

##### Detached connection.

People spoke about some of the difficulties with their experience of receiving support online and this feeling more detached.

*I find there is something detached about online treatment in that it doesn't feel like you're really connected to the other person because at the end of the call, it's just you in the same room as you live day to day. Also, for a while my therapist used a false background that left me feeling really uneasy as it made the session feel somehow less real/professional. [M1, AN]*

For some, there was a feeling of online treatment being less safe because of not having a confidential space at home to express themselves freely. For a smaller number of participants, their anxiety about telephone or video calls meant they felt unable to engage in this support at all. Frustrations with the quality of the internet connection were an additional hindrance to participants being able to engage fully.

*I dont feel that I can talk over video call whilst in the house with partner and children within hearing distance [F101, BED]*

Many participants spoke about the connection with the therapist as feeling less personal and more detached in comparison to face-to-face contact. This detached connection became a barrier to some participants being able to engage fully. For a few participants, there was more opportunity to hide or not disclose important information.

*Now video meetings have started.. but I'm far less open in these sessions and hide … what has been going on. [F13, AN-bp]*

In contrast to this detached connection, there were many participants who commented on their experience of having a strong therapeutic relationship, which allowed them to feel safe, secure, able to connect, and to feel heard and believed.

*Weekly connection. Feeling heard Feeling believed [F17, AN].*

For participants with a positive therapeutic relationship, this seemed to protect them from the feeling a detached connection.

##### Ease of access.

The move to online treatment made treatment more accessible for some participants. Online treatment reduced lengthy travel times due to the large geographical regions covered by many specialist eating disorder services.

*We have moved to online sessions but it is good not to have to travel an hour each way. [F62, BN]*

The online aspect also reduced some of the anxiety-provoking barriers and allowed a few participants to feel more able to seek help.

*Being able to have regular contact with medical professionals via telephone has made it much easier for me to seek help, as it feels much lower commitment, and gives me far less anxiety, than seeking out face-to-face treatment. [F102, BN]*

#### Theme 8: loss of support

Participants described their experience of support during the pandemic as being of a lesser quality than their usual support. For many, the typical structure and expectations of active treatment were lost, or paused, and replaced with a less formal “check-in” chat.

*I have been unable to go to my appointments, I have had some telephone sessions but they have not been as productive. [F116, AN]*

Other participants spoke about a loss of treatment support. Treatment was either delayed or stopped for many. Some services were closed and there was a sense of loss at not receiving support in a time of need.

*Clinics have closed. No face to face appointments. No-one to talk to. [M23 BN]*

Some participants described the loss of valued support from their GP as well as their specialist ED support.

*Do not get to see my GP any more. He was my lifeline and my rock. [SM22, AN]*

Another factor preventing many participants from accessing treatment was strong feelings of guilt about taking up NHS time and not wanting to bother NHS services during the pandemic. One participant described feeling “*like a grotesque burden” [F39, AN].* Some participants expressed feeling undeserving of help.

*I've definitely felt guilty using NHS services, i've also felt undeserving of counselling/therapy sessions. [M45, OSFED]*

The hesitancy in seeking help also applied to participants who had experienced a relapse of their ED during the pandemic; participants spoke about waiting to ask for support until the end of lockdown.

*I was doing well but Covid and lockdown made me think again about food. I am considering going back to the Dr's to request treatment now that we are seeing the end of lockdown, as I wish to go back to coping with my issues. [M66, BED]*

In contrast, a few participants spoke about the loss of support being an opportunity for them to take more responsibility for helping themselves. For participants who spoke about having the knowledge and skills to enable themselves to get back on track, they were able to put their experience of previous therapy into practice allowing themselves to be their own therapist, despite not having access to the support of services during this time period.

*It gave me a sense of direction on how to help myself as at the end of the day it's myself that needs to be able to cope with my own mental well being. [M38, AN]*

The finding that a small number of participants were able to take positive steps towards looking after their mental wellbeing reflects our quantitative finding that a minority of participants reported symptom improvement or symptom maintenance.

## Discussion

In a tripartite approach, the present study sought to 1) extend and crystallise former reports around the impact of COVID-19 on people with eating disorders (EDs); 2) examine emotion regulation processes and their predictive value as regards to psychological wellbeing, ED symptomatology and symptom change during the pandemic; and [3] explore the experiences of those receiving online psychological treatment. To this end we collected qualitative and quantitative data from a large sample of people with an ED. We will discuss each goal in turn, hoping that through exploring these experiences, we will elucidate pathogenic influences of the pandemic, protective interpersonal mechanisms, and factors that impede and facilitate the efficacy of remote interventions during a time of crisis.

### Worsening of symptoms during the pandemic

As governments adapt to managing the threat of coronavirus, the strictest lockdown measures are hoped to slowly lift. People establish new normalities, but lessons from history and trauma studies suggest that the psychological impact of collective and individual trauma will continue for a long time to come [2, 36, 37]. Accordingly, even though our data was collected at a point (June–July, 2020) after the severest quarantine measures in the UK, most of our participants still reported a worsening in ED symptomatology since before the pandemic. It is likely that increased support will be required for a long time, so research is crucial to better understand how to help these individuals.

To assist clinicians in identifying the most appropriate targets for intervention, we explored the relative importance of factors identified in preliminary research [14] in terms of their impact on the individual’s ED. Across all four diagnoses, the most marked impacts were related to difficulties with emotions, disruption to routine, and changes in physical activity schedule, all concerns which had been previously highlighted in the aforementioned literature. There was, however, striking variability between participants, a heterogeneity which might depend on situational factors and/or diagnosis, and which should be considered by clinicians.

One noticeable difference in the quantitative data suggested that people with AN or OSFED ranked triggering messages on social media as more detrimental than did people with BED. Much has been written around the association between social media use, thin idealisation, disordered eating and eating disorders [38–40]. Thin idealisation online has manifested at the present time in messages around avoiding quarantine weight gain, messages which have been suggested to be dangerous to people with EDs [18, 41]. The impact of such pernicious messages was reflected in our qualitative data, which likewise found that triggering messages led to increased pressure to eat healthily, to exercise, and to lose weight, which exacerbated ED behaviours. In accordance with the quantitative data, participants who described being triggered by social media were generally those with AN, some of whom were triggered even by ostensibly positive messages. Notably, some participants mentioned a pressure towards self-improvement and productive use of lockdown. Concerns have been raised about people with pathological perfectionism in the context of the vanishing divide between work and home, the tendency for stressful situations to increase the drive to achieve mastery, and the difficulty of these individuals to adjust self-standards despite exceptional circumstances [42]. These concerns have also been raised in the context of EDs [18], where additional risks around “self-improvement” messages lie in the social reinforcement of thin idealisation, diet and exercise. In so far as participants with AN and with OSFED appeared more affected by triggering social media, it is interesting that some studies suggest perfectionism is associated less with bingeing than with other types of eating pathology, such as restriction and purging [43–45]. In accordance with its status as a lesser known, stigmatised and misunderstood eating disorder [46–48], the particular social media usage of individuals with BED is unknown; the different impact of social media exposure on people with BED in the present study is, as such, an intriguing avenue for future research.

### The role of emotion regulation

As predicted, emotion regulation was a significant predictor of our wellbeing outcomes. This was particularly the case for general mental distress (DASS scores) as an outcome variable, for which emotion regulation explained almost 50% of the variance. This is consistent with the first part of our analysis, namely the highlighting of ‘difficult emotions’ as one of the most impactful pandemic factors on ED symptomatology, and is wholly redolent of the theoretical stance that negative emotions and inadequate regulation strategies are central to the aetiology and maintenance of the whole spectrum of ED psychopathology [22–24, 49, 50].

Amongst all DERS subscales, lack of emotional clarity, lack of access to emotion regulation strategies, and non-acceptance of emotions were found to be the strongest significant predictors of mental distress. As pertains to this first predictor, lack of emotional clarity is characteristic of alexithymia, a personality trait characterised by an inability to identify and describe emotions. Alexithymia occurs at higher rates both in people with an ED [26] and populations more vulnerable to EDs, such as autistic people [51, 52], such that some query whether it might mediate this increased vulnerability [53]. Clarity and awareness of emotions are thought to occur early in the process of emotion regulation, such that the ability to identify feelings helps determine which strategy to use to regulate unpleasant emotions [54, 55]. Interestingly, lack of access to emotion regulation strategies was also a strong predictor of distress during the pandemic. It is possible that these two are linked, and that helping patients identify their feelings with more clarity could help them match specific negative emotions to one in an arsenal of adaptive strategies. Worryingly, maladaptive and avoidant strategies, such as ED behaviours, alcohol use, self-medicating and self-harm were common responses to difficult emotions in the pandemic, as in previous reports [11]. Unfortunately, these kind of maladaptive emotion regulation strategies are activated easily and automatically, while adaptive strategies require conscious activation [56].

Despite this, our qualitative analysis reflected that some participants *were* still able to identify and apply adaptive strategies in their lives: these included accessing social support, setting appropriate boundaries for self-care and protection, and adding adaptive strategies in the form of new structures and reinforcing activities, as implicitly aligned with behavioural activation approaches to depression [57]. Only two studies have examined the coping activities engaged in by people with EDs at this time: consistent with the importance of setting new structures and enjoyable activities highlighted by our participants, these previous studies found that among the most helpful activities for people with AN and BN were establishing routines, day planning and scheduling pleasant activities [12, 13], in addition to time with family, yoga and gentle exercise.

Non-acceptance of emotions was not explicitly mentioned by participants in our qualitative analysis but was however associated with greater distress in our quantitative analysis. The ability to accept negative emotional experiences without judgement has consistently been associated with improved wellbeing [58–60], and thus-far unpublished data collected cross-culturally by the lead author [61] found benefits of emotion acceptance above active regulation strategies (like reappraisal) during the pandemic.

Together, our findings – the increase in negative emotions; the relationships between negative emotions and ED psychopathology (as reflected numerically, statistically and explicitly stated by participants), and between regulation deficiencies and negative affect; the reported benefits from adaptive regulation strategies, and the statistical support for the advantages of emotion acceptance – highlight several clear points for therapeutic intervention to intercede the cyclic maintenance of ED psychopathology by negative emotions and maladaptive responses [22–24, 49, 50]. While clarification of emotional experience may be a crucial first step, this might be followed by attempts to increase accessibility of existing adaptive regulation strategies and develop new ones, including emotion acceptance, in response to distress. Dialectical Behaviour Therapy (DBT) for BED and BN was developed in response to patients who do not always benefit from Cognitive Behaviour Therapy (CBT)-focused treatments [62], but this and other third-wave interventions such as acceptance and commitment therapy (ACT), compassion-focused therapy (CFT) and mindfulness have wider potential in the treatment of people with EDs [63]. Their relevance in this context comes from the fact that in contrast to pure CBT which attempts to prevent strong negative emotions being activated, third wave interventions are response-focused [64]: centred around accepting, managing and finding adaptive ways to moderate the experience of strong emotions when they arise. The existing evidence suggests that CBT may be more effective than third wave therapies [63–65], but these analyses all focus on a time prior to Covid-19. Whether third wave, response-focus therapies might be useful at a time when people have limited control over the antecedents of negative emotion is an intriguing query for future researchers. Adaptions to treatment paradigms in the current climate must, however, be made with caution and based on further experimental scrutiny.

### Experience of online treatment

The third strand of our analysis focused on exploring the responses of those within our UK-based sample who had been offered remote psychological interventions. Very little is presently known around the efficacy of online treatment during the present crisis, but some preliminary concerns raised by individuals and their families [10] were ratified in our analysis. Where participants in the previous study suggested that therapeutic contact online would certainly “lack something” [10], our participants also reported a feeling of “detachment”; a diminishment of the expected and desired connection with the clinician, partly due to practical difficulties, such as internet connectivity and participants being unable to find a safe and private place within their homes. Interestingly however, our participants with pre-existent therapeutic relationships seemed partly protected from this issue, and reported some benefits from maintained therapeutic contact. Likewise, the previous study [10] raised concerns around the loss of structure, the loss of more active therapeutic activities, such as homework, and the loss of accountability, for instance related to weigh-ins – concerns aligned with the comments of our participants. This allowed some of our participants to engage unchecked in ED behaviours (“I have no one around to keep an eye on me”), consistent with the ambivalence that people with EDs often feel around treatment and recovery [66, 67]. It is important to recognise that these data, and that of the previous study [10], are time-sensitive. At the point of our data collection in June–July 2020, NHS services had recently undergone mass reorganisation and deployment of staff. Though the NHS remains in a state of flux and differences are still present across localities, the importance of “business as usual” and clear, practical guidance around how to establish the same level of excellence have since been disseminated [18, 30, 31, 68, 69]. For example, with reference to issues around engagement, motivation and openness, clinicians have been recommended to thoroughly and openly explore the patient’s previous experiences of and attitudes towards remote treatment; to discuss and plan the practical implementation of the therapy and develop contingency plans for issues such as connection problems; to use the full power of audiovisual technology to create openness and connection; and to, as much as possible, involve the patient as an active collaborator in therapy [18, 30, 31].

While online treatment was found to be lesser than face-to-face treatment on many aspects, some participants felt that this form of therapy demanded a “lower commitment” and was less anxiety-provoking, which confirms previous suggestions that telehealth interventions might be favoured by people with EDs due to feeling less self-conscious about their body and more in control [70]. It is however important to note that for some, using video may be upsetting, so a recent paper recommended starting with audio if the patient has concerns about seeing their own image, and/or asking them to not look at parts of their own image they find distressing, although the therapist and patient should ideally work together to accept switching to video [31]. Another important aspect concerned the convenience of online treatment which reduced lengthy travel times – currently considered as a barrier to successful treatment [71, 72]. The positive appraisals around convenience and ease of access, found in our participants and in those of the previous study [10] were actually identified as advantages of telehealth interventions prior to Covid-19 [29, 73, 74] and suggest a future for online treatment beyond the COVID-19 pandemic.

Importantly, we observed huge variation across participants’ experiences, captured by the following quote “Perhaps acknowledgement of how clients differ would also help, rather than a blanket here’s-how-we’re-doing-it-now approach” [M61, BED]. Given comments from participants struggling with achieving space and privacy from their families, this is one among other circumstantial factors likely to have substantial impact, and which requires consideration and possible tailoring of interventions. Further investigation of the factors relating to the efficacy and acceptability of remotely-delivered evidence-based treatments is an important goal.

One last, worrying issue which emerged in our analysis was around the loss of access to other primary healthcare infrastructure (for instance, GPs) and the reticence that many participants reported around seeking help. Many reported feelings of guilt and being undeserving of treatment, which may link to the lack of self-worth underlying ED symptomatology, where low self-esteem becomes intertwined in an over-evaluation of weight and shape [75]. Internalised stigma may also contribute to these feelings [76]. However, it is possible that this is, in addition, an unintended consequence of governmental measures and communications aimed at reducing transmission and preserving medical resources to cope with Covid-19. Worrying evidence is emerging that despite increased reports of domestic violence, self-injury and suicidality, fewer individuals than would be typically expected are seeking formal help from health professionals [77]. Concerns that mental ill-health might be perceived as less important than physical health [78] seemed to be borne out in our participants. To some extent, solutions may be found in the possibility of self-referrals as an option, and in increasing awareness of the availability of online support groups, as access to these groups was identified as helpful by some participants. Research exploring the impact and perception of public health announcements may, however, be vital in ensuring that those who need to do seek help.

### Limitations

While this study contributes to understanding the effect of COVID-19 on a population with pre-existent mental illness, there are several important limitations to consider. First, the time sensitivity of the study necessitated speedy and convenient recruitment of participants through social media and Prolific. While this allowed us to collect a larger and more sex-balanced sample than previous research on COVID-19 and EDs [9, 14], we relied on self-reported diagnoses. Although the EDEQ confirmed that average eating psychopathology was in the clinical range, it would have been beneficial to confirm diagnoses, which may have been especially important in our attempts to contrast different ED diagnoses. Moreover, our sample was self-selected and over 18, meaning that it is possible that it was biased towards people who may have more severe eating disorders, or who had not been able to use treatment to make change. Our sample indeed had a longer than average duration of illness in comparison to recent estimates for untreated eating disorders [79]. Therefore, it is possible that this study’s sample may not be a fully generalisable representation of the entire eating disorder population, including of the younger population.

Further issues with our sample included underrepresentation of minority ethnic groups, and unequal ratio of female to male participants. Particular efforts had been made to recruit this often forgotten population, and were partially achieved in that we recruited 76 men with EDs. However as the distribution of men and women differed across specific ED diagnoses and the male sample was still comparatively underpowered, we were forced to abandon our hope to compare male and female participants with each specific type of ED pathology. Consequently, it is possible that differences between ED diagnoses, for instance in the greater importance of social media triggers for participants with AN and OSFED than for participants with BED, might be a factor of the different representation of women and men within these groups. Here, again, the paucity of data on ED in males creates the danger that in the present time, too, men with EDs may be less understood.

Finally, our cross-sectional design precludes any conclusions around the causal links between emotion regulation strategies and our wellbeing outcomes. Indeed, while extant literature supports the claim that adaptive emotion regulation strategies (such as emotion acceptance) are associated with better mental health [56, 60], it is possible that high levels of distress in our study could have led to more difficulties with emotion regulation. Longitudinal studies on the impact of emotion regulations strategies during COVID-19 could help better inform the direction of the effect.

### Implications

Our findings point to several clinical implications for healthcare professionals to consider. With the likelihood that services will continue to work remotely for some time, it is crucial that services continue to deliver the most effective evidence-based treatments with its usual structure.

Our paper highlights some of the ways that reduced emotion coping skills, along with changes to routine, social contact, and living environments have impacted on people’s EDs and therefore may be important areas to address within treatment. In terms of routine, we suggest that clinicians could use techniques from behavioural activation [57], which equips patients with skills to structure their day with activities which give a sense of achievement, a sense of enjoyment, and a sense of social connection.

Our findings also highlighted the importance of setting appropriate boundaries around exposure to media, alcohol, eating and exercise routines, to encourage self-care in each of these areas. These implications for treatment are consistent with other recommendations from the literature on ED during the pandemic, which have highlighted the importance of increasing value activities, social contact and placing boundaries to encourage healthy behaviours relating to eating and exercise behaviours [10, 18, 69].

For patients who use their ED as a way of coping with difficult emotions and distress, treatment may also incorporate emotion regulation skills as referenced within CBT-ED approaches [75] and discussed in further detail within DBT treatment [62]. This may include helping people to identify and label their emotions, recognise the function of their emotions, and learn adaptive strategies for coping with distressing emotions, such as emotional acceptance.

Healthcare professionals may also want to consider exploring the patient’s experience of online treatment and identify potential therapy-interfering behaviours, such as our participants who identified a tendency to hold back from being open and honest in treatment sessions. A collaborative approach would allow for discussion of how potential barriers can be managed. Our findings show that the therapeutic relationship may protect from feelings of disconnection; an openness to gather in-session feedback may help patients continue to feel supported and understood, despite the connection being remote. Finally, there was a wide variation in patients’ responses across our qualitative themes and it is important to tailor the treatment approach to an individual’s formulation, whilst still adhering to the evidence-based approach.

## Conclusion

Through a mixed-method tripartite approach, we showed that our self-selected participants with EDs have been strongly affected during the COVID-19 pandemic, and that difficulties regulating emotions was a significant predictor of their well-being. We also showed that while online treatment had some beneficial aspects, more work is needed to fully understand its impact on patients’ mental health. We recommend that future research further explore the benefits of working on emotion regulation strategies as part of treatment in a post-pandemic world at a time when people have limited control over the antecedents of negative emotions.

## Supplementary Information

**Additional file 1.**

## Data Availability

The datasets used and/or analysed during the current study are available from the corresponding author on reasonable request.

## Abbreviations

AN: Anorexia Nervosa
ARFID: Avoidant/Restrictive Food Intake Disorder
BED: Binge Eating Disorder
BN: Bulimia Nervosa
CBT-E: Enhanced Cognitive Behaviour Therapy
CBT-ED: Eating Disorder Focused Cognitive Behaviour Therapy
DASS: Depression, Anxiety and Stress Scale
DERS: Difficulties in Emotion Regulation Questionnaire
ED: Eating Disorder
EDEQ: Eating Disorder Examination Questionnaire
MANTRA: Maudsley Model of Anorexia Treatment in Adults
NHS: National Health Service
SSCM: Specialist Supportive Clinical Management

## Footnotes

1 We removed participants who reported having been given two diagnoses such as AN and BN because the DSM-5 precludes dual diagnosis. According to the DSM-5, if the person is significantly underweight, they would present with AN. As such, a diagnosis of AN would always trump a diagnosis of BN, even if the person binges and purges (in that case they would be diagnosed with AN binge purge type). We aimed to clarify with our participants these dual diagnoses when possible. This was not possible for our social media sample which was anonymous, so these four participants were removed from the analysis
