# Supplementary material for: The impact of the COVID-19 pandemic on individuals with eating disorders: the role of emotion regulation and exploration of online treatment experiences
Source: J Eat Disord. 2021 Jan 12;9:10. doi: 10.1186/s40337-020-00362-9 (PMC7802411; doi:10.1186/s40337-020-00362-9)
Supplement: Supplementary file 1 — Additional file 1. [file 40337_2020_362_MOESM1_ESM.docx]

**Supplementary Materials**

*Table of contents*

- Supplementary item 2: Qualitative survey
- Supplementary item 3: Correlations between pandemic factors
- Supplementary item 4: Reported change in symptoms as split per diagnosis
- Supplementary items 5: Relative importance of pandemic factors to exacerbation of ED symptomatology
- Supplementary item 6: Regression models split by diagnosis category

**Supplementary item 1: Details of the measures used**

### In order of completion, the details of our measures are as follows:

### **EDEQ**. As diagnoses could not be clinically verified, the EDEQ served both to validate self-reported diagnoses and as an outcome measure of eating psychopathology during the pandemic. Group scores were comparative with clinical norms from previous literature (36) and above norms seen in non-clinical samples (37). The EDEQ total score has strong internal consistency (α=.90) (38) which was precisely confirmed in our sample (α=.90).

### **Difficulties in Emotion Regulation Scale, Short Form.** The DERS-SF is an 18-item scale assessing difficulties with emotion regulation. This measure uses a 5-point Likert scale, with answers ranging from ‘Almost Never’ (1) to ‘Almost Always’ (5), on six subscales with three items each: Strategies, Non-acceptance, Impulse, Goals, Awareness, and Clarity. Higher scores are found to indicate difficulties with emotional regulation. The DERS-SF has been shown to have good reliability (35,39), which was confirmed in our sample (total: α = .90, Strategies: α = .85, Non-acceptance: α = .81, Impulse: α = .89, Goals: α = .88, Awareness: α = .77, and Clarity: α = .84).

### **Qualitative Survey (see supplementary materials, 1).** With a free text item, participants were first asked to explain, in their own words, the effect of the pandemic on their ED symptoms, and to indicate this on a 7-point Likert scale ranging from Much worse (7) to Much better (1). On a scale of 0-100, participants then rated the extent to which their ED symptoms had been affected by each of the nine aspects identified by Branley-Bell and Talbot (2020): disrupting to living situation (e.g. being better/less able to hide behaviours due to being alone at home/living with family); change in social support (e.g. more/less support from friends and family); change to physical activity (e.g. change in exercise routine due to gym closure/more time at home to exercise); change to food availability (e.g. shortage of ‘safe’ food; stockpiling; need to buy healthier food); exposure to triggering messages (e.g. social media with many people posting pictures or comments about food or exercise, or post about how to look good when video conferencing); disruption to routine (e.g. lack of routine; loss of a job); emotions (e.g. anxiety around the unknown situation, stress of having to multitask between work and childcare); change in treatment (e.g. premature discharge, change to online, feeling guilty for taking up NHS time); and physical heath (e.g. fearing to get/having Covid-19, a loved one having Covid-19, etc). They were able to expand on each aspect with qualitative comments, and a summary item allowed them to provide and indicate the impact of any other factors that were missed. We then asked about their coping mechanisms prior and during the pandemic, and whether they identified any of these as particularly helpful or, in contrast, harmful. Participants were asked if they were currently receiving psychological therapy, and if so, what kind of therapy that was (face to face, for instance in-patient, day-patient or out-patient treatment; online therapy; or telephone therapy; those who selected remote therapy were then asked if this was CBT-ED, MANTRA, SSCM, psychodynamic therapy, family-based therapy, guided self-help, brief wellbeing checks, or something else). Participants were asked to explain in their own words their experience of treatment during the pandemic, and if they received online treatment, what had been helpful and unhelpful about it and what could improve it. Participants who were not receiving therapy were given the opportunity to explain why, and finally, all participants were given the opportunity to add any final comments on the impact of the pandemic on their ED, coping behaviours, treatment, and their lives in general.

**The Depression, Anxiety and Stress Scale.** The DASS incorporates 21 statements corresponding to depression, anxiety and stress experienced over the last week (7 items each). Participants responded to each item on a 4-point Likert Scale ranging from ‘Did not apply to me at all’ (0) to ‘Applied to me very much, or most of the time’ (3). Summed scores are doubled to correspond with the original, 42-item version, and higher scores reflect greater depression, anxiety and stress. The present study used only the total score. The scale is popularly used cross-culturally and boasts good psychometric properties (40,41), which were confirmed herein with high internal consistency of the total score (α = .92).

**Supplementary item 2: Qualitative survey**

The qualitative survey completed by participants was presented as follows. For clarity, the few quantitative items (related to rating the impact of the pandemic on the participant’s ED, and the impact of specific aspects of the pandemic on their ED) are preserved here in the way that they were originally presented within the qualitative questionnaire.

Qualitative items are marked out in blue for clarity, quantitative items in green.

----

The following questions are about the specific impact of the Covid-19 pandemic (henceforth ‘the pandemic’) on your eating disorder symptoms, that is, the behaviours, emotions and thoughts related to your ED. The pandemic has impacted people in many different ways, and different things or aspects about it may have had specific impacts on you. We’d love to understand this, and so these questions will try to explore in more detail how the pandemic has affected you.

The more you write, the better we can understand the impact of the pandemic for you, so please do not feel that you have to hold back. You are free to write as much or little as you like.

***(Free text response)*** Could you please tell us in your own words how your eating disorder symptoms have been affected by the pandemic?

***(Quantitative outcome measure used in regression)*** Generally, during the pandemic, do you think your ED symptoms have been....

- Much worse
- Moderately worse
- Slightly worse
- No change
- Slightly better
- Moderately better
- Much better

***(PAGE BREAK)***

We are now going to talk about several different aspects of the pandemic situation.

For each of the following aspects, could you please tell us how important each one has been in relation to your ED? You can do this, for each item, by moving the slider along a scale of 0 to 100 (0 being not important at all, and 100 being very important to your situation).

There is no right or wrong answer, just tell us what was you think has played a role in your change in symptoms. For each one, there's an opportunity for you to expand on how this aspect of the pandemic has affected you. You'll get a little reminder if you forget to move the slider on any of the items, but you don't need to write something extra for each one (or any of them). We encourage you to write as much or as little as you like; we are always happy with whatever you can give us.

So here is the first one:

1. DISRUPTION TO LIVING SITUATION (e.g. being better/less able to hide behaviours due to being alone at home/living with family).

***(Quantitative)*** How important has this aspect of the pandemic been in relation to your ED? (0 not important at all, 100 being very important)

***(Qualitative)*** Do you want to expand on this? Please write it in the box?

1. CHANGE IN SOCIAL SUPPORT (e.g. more/less support from friends and family).

***(Quantitative)*** How important has this aspect of the pandemic been in relation to your ED? (0 not important at all, 100 being very important)

***(Qualitative)*** Do you want to expand on this? Please write it in the box?

1. CHANGE IN PHYSICAL ACTIVITY (e.g. change in exercise routine due to gym closure/more time at home to exercise).

***(Quantitative)*** How important has this aspect of the pandemic been in relation to your ED? (0 not important at all, 100 being very important)

***(Qualitative)*** Do you want to expand on this? Please write it in the box?

1. **CHANGE IN FOOD AVAILABILITY** (e.g. shortage of ‘safe’ food; stockpiling; need to buy healthier food).

***(Quantitative)*** How important has this aspect of the pandemic been in relation to your ED? (0 not important at all, 100 being very important)

***(Qualitative)*** Do you want to expand on this? Please write it in the box?

1. **EXPOSURE TO TRIGGERING MESSAGES**(e.g. Social media with many people posting pictures or comments about food or exercise, or posts about how to look good when video conferencing).

***(Quantitative)*** How important has this aspect of the pandemic been in relation to your ED? (0 not important at all, 100 being very important)

***(Qualitative)*** Do you want to expand on this? Please write it in the box?

1. **DISRUPTION TO ROUTINE**( e.g. lack of routine; loss of a job).

***(Quantitative)*** How important has this aspect of the pandemic been in relation to your ED? (0 not important at all, 100 being very important)

***(Qualitative)*** Do you want to expand on this? Please write it in the box?

1. **EMOTIONS**(e.g. anxiety around the unknown situation, stress of having to multitask between work and childcare).

***(Quantitative)*** How important has this aspect of the pandemic been in relation to your ED? (0 not important at all, 100 being very important)

***(Qualitative)*** Do you want to expand on this? Please write it in the box?

1. **CHANGE IN TREATMENT**(e.g. premature discharge, change to online, feeling guilty for taking up NHS time).

***(Quantitative)*** How important has this aspect of the pandemic been in relation to your ED? (0 not important at all, 100 being very important)

***(Qualitative)*** Do you want to expand on this? Please write it in the box?

1. **PHYSICAL HEALTH**(e.g. fearing to get/having COVID-19, a loved one having COVID-19 etc).

***(Quantitative)*** How important has this aspect of the pandemic been in relation to your ED? (0 not important at all, 100 being very important)

***(Qualitative)*** Do you want to expand on this? Please write it in the box?

1. Is there anything else about the pandemic situation that has affected your ED?
   ***(Quantitative, qualitative)*** If so, please write down what that thing is in the box, and move the slider along to reflect how important has this aspect of the pandemic been in relation to your ED (0 not important at all, 100 being very important).

***(PAGE BREAK)***

When it comes to difficult feelings or things in their lives, people cope in different ways. In this section we are interested in the strategies you have used or are still using to cope with this situation.

***(Free text response)*** What would you normally do to cope with difficulties in your life?

***(Free text response)*** How have your coping strategies been affected by the Covid-19 pandemic?

***(Free text response)*** Can you tell us about any coping strategies which have been particularly helpful during the pandemic?

***(Free text response)*** Have any of your coping strategies been harmful in any way? Can you tell us a little more about this?

***(PAGE BREAK)***

In this last section, we are interested in how the pandemic has affected any psychological treatment that you may be receiving or have received.  Have you experienced psychological treatment for your eating disorder during the pandemic (i.e. since Mid-March)?

- Yes - No

If so, what was your treatment during the pandemic? Tick any that apply:

- Face to face (if so, please say whether it was inpatient, day-patient or out-patient treatment)
- Online
- Telephone

***(if online treatment selected)***

Could you please specify what type of **online** treatment you received?

- CBT-E
- MANTRA
- SSCM
- Psychodynamic therapy
- Family based treatment
- Guided self-help
- Brief well-being checks
- Other ***(free text response)***

***(free text response)*** Can you tell us about your experience of **online treatment** during the pandemic?

***(free text response)*** What have been the helpful aspects of **online treatment** been in your experience?

***(free text response)*** What have the unhelpful aspects of online treatment been in your experience?

***(if face to face or telephone selected)***

***(free text response)*** Can you tell us about your experience of treatment during the pandemic?

***(all)***

***(free text response)*** What do you think could make **online treatment** better?

***(Free text response)*** Is there anything else you want to say about the impact of the pandemic on your eating disorder, your coping behaviours, any treatment you may or may not be receiving, or your life in general before we move to the next part of the study?

Would you want to tell us why you are not currently receiving treatment? 

For example, you may not be ready for change yet, you may be awaiting treatment, or you may have other reasons. It might be that Covid-19 has affected your feelings about asking for help. 

***(Free text response)*** Please write anything that will help us understand your situation better.

***(Free text response)*** Is there anything else you want to say about the impact of the pandemic on your eating disorder, your coping behaviours, any treatment you may or may not be receiving, or your life in general before we move to the next part of the study?

**Supplementary item 3: Correlations between pandemic factors**

Having selected only those participants who reported a deterioration in their ED symptoms, we plotted non-parametric correlations between variables. A single asterisk (*) indicates correlations significant at p < .05; double asterisks indicate correlations significant at p < .01; triple asterisks indicate correlations significant at or under p = .001. Here, results displayed for **all participants pooled (n = 172)**, cells are colour-coded by the strength of correlation coefficients as per the following key:


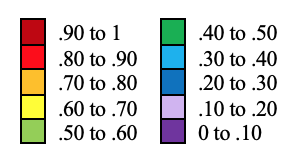


|  | Disruption to living situation | Change in social support | Change in physical activity | Change in food availability | Exposure to triggering messages | Disruption to routine | Emotions | Change in treatment | Physical health concerns |
| --- | --- | --- | --- | --- | --- | --- | --- | --- | --- |
| Disruption to living situation |  |  |  |  |  |  |  |  |  |
| Change in social support | **.266 ***** |  |  |  |  |  |  |  |  |
| Change in physical activity | **.178 *** | .125 |  |  |  |  |  |  |  |
| Change in food availability | .116 | .107 | **.161 *** |  |  |  |  |  |  |
| Exposure to triggering messages | **.283 ***** | **.317 ***** | **.317 ***** | .013 |  |  |  |  |  |
| Disruption to routine | .129 | .023 | **.192 *** | **.184 *** | **.248 ***** |  |  |  |  |
| Emotions | **.220 **** | **.329 ***** | **.251 ***** | **.225 **** | **.282 ***** | **.371 ***** |  |  |  |
| Change in treatment | **.227 **** | **.335 ***** | **.156 *** | .130 | **.256 ***** | .047 | **.256 ***** |  |  |
| Physical health concerns | **.239 ***** | **.243 **** | **.255 ***** | **.223 **** | **.213 **** | .130 | **.367 ***** | **.268 ***** |  |


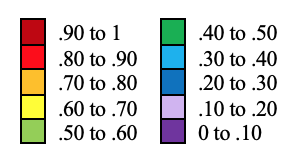


We then checked to be sure that correlations held for individual diagnostic groups. These are displayed as follows for participants with **anorexia nervosa (n = 70):**

|  | Disruption to living situation | Change in social support | Change in physical activity | Change in food availability | Exposure to triggering messages | Disruption to routine | Emotions | Change in treatment | Physical health concerns |
| --- | --- | --- | --- | --- | --- | --- | --- | --- | --- |
| Disruption to living situation |  |  |  |  |  |  |  |  |  |
| Change in social support | .173 |  |  |  |  |  |  |  |  |
| Change in physical activity | .099 | .100 |  |  |  |  |  |  |  |
| Change in food availability | .143 | .175 | **.258 *** |  |  |  |  |  |  |
| Exposure to triggering messages | .118 | **.306 *** | **.382 ***** | .238 |  |  |  |  |  |
| Disruption to routine | **.269 *** | .060 | **.251 *** | .212 | **.264 *** |  |  |  |  |
| Emotions | **.352 **** | **.331 **** | **.334 **** | .218 | **.506 ***** | **.444 ***** |  |  |  |
| Change in treatment | **.325 **** | .228 | **.254 *** | **.276 *** | .224 | .084 | **.300 *** |  |  |
| Physical health concerns | **.363 **** | **.365 **** | .133 | **.270 *** | .089 | .173 | **.308 *** | **.270 *** |  |


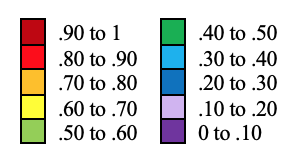


Participants with **bulimia nervosa (n = 38):**

|  | Disruption to living situation | Change in social support | Change in physical activity | Change in food availability | Exposure to triggering messages | Disruption to routine | Emotions | Change in treatment | Physical health concerns |
| --- | --- | --- | --- | --- | --- | --- | --- | --- | --- |
| Disruption to living situation |  |  |  |  |  |  |  |  |  |
| Change in social support | **.317** |  |  |  |  |  |  |  |  |
| Change in physical activity | .273 | -.075 |  |  |  |  |  |  |  |
| Change in food availability | .001 | .056 | .059 |  |  |  |  |  |  |
| Exposure to triggering messages | **.395 *** | **.388 *** | **.394 *** | -.059 |  |  |  |  |  |
| Disruption to routine | .052 | -.209 | .231 | **.385 *** | .077 |  |  |  |  |
| Emotions | .077 | .178 | .117 | **.438 **** | .050 | .224 |  |  |  |
| Change in treatment | .209 | .264 | .220 | .106 | .021 | -.207 | .131 |  |  |
| Physical health concerns | .049 | .265 | .254 | .159 | .097 | .066 | **.637 ***** | .084 |  |


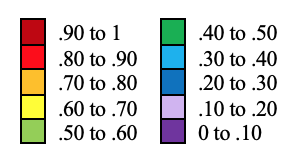


Participants with **binge eating disorder (BED) (n = 40):**

|  | Disruption to living situation | Change in social support | Change in physical activity | Change in food availability | Exposure to triggering messages | Disruption to routine | Emotions | Change in treatment | Physical health concerns |
| --- | --- | --- | --- | --- | --- | --- | --- | --- | --- |
| Disruption to living situation |  |  |  |  |  |  |  |  |  |
| Change in social support | **.411 **** |  |  |  |  |  |  |  |  |
| Change in physical activity | .279 | .186 |  |  |  |  |  |  |  |
| Change in food availability | **.361 *** | **.409 *** | .286 |  |  |  |  |  |  |
| Exposure to triggering messages | **.411 **** | .244 | .090 | .017 |  |  |  |  |  |
| Disruption to routine | .261 | -.192 | .009 | .050 | **.379 *** |  |  |  |  |
| Emotions | **.318 *** | **.446 **** | .171 | .315 | .292 | .258 |  |  |  |
| Change in treatment | **.453 **** | **.549 ***** | .049 | .192 | **.385 *** | .113 | **.392 *** |  |  |
| Physical health concerns | **.409 **** | .252 | .286 | **.383 *** | **.466 **** | **.330 *** | **.382 *** | **.396 *** |  |


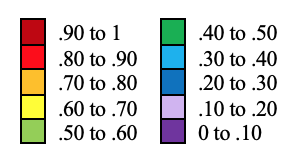


Participants with **OSFED/EDNOS (n = 24):**

|  | Disruption to living situation | Change in social support | Change in physical activity | Change in food availability | Exposure to triggering messages | Disruption to routine | Emotions | Change in treatment | Physical health concerns |
| --- | --- | --- | --- | --- | --- | --- | --- | --- | --- |
| Disruption to living situation |  |  |  |  |  |  |  |  |  |
| Change in social support | .079 |  |  |  |  |  |  |  |  |
| Change in physical activity | .063 | .188 |  |  |  |  |  |  |  |
| Change in food availability | .027 | -.129 | -.008 |  |  |  |  |  |  |
| Exposure to triggering messages | .328 | .362 | **.434 *** | -.380 |  |  |  |  |  |
| Disruption to routine | -.182 | .613 | **.412 *** | -.039 | .288 |  |  |  |  |
| Emotions | -.007 | **.469 *** | .299 | -.164 | .131 | **.562 **** |  |  |  |
| Change in treatment | -.366 | .220 | .140 | -.241 | .426 | .207 | .009 |  |  |
| Physical health concerns | .011 | -.101 | **.592 **** | .026 | .257 | -.160 | -.084 | .181 |  |

**Supplementary item 4: Reported change in symptoms as split per diagnosis**

The number and percentage of participants from each diagnostic group who reported exacerbation, improvement or no change in ED symptomatology.

|  | AN  n (%) | BN  n (%) | BED  n (%) | OSFED  n(%) |
| --- | --- | --- | --- | --- |
| Much worse | 23 (25.3) | 9 (19.6) | 11 (25.0) | 7 (26.9) |
| Moderately worse | 30 (33.0) | 22 (47.8) | 17 (38.6) | 12 (46.2) |
| Slightly worse | 17 (18.7) | 7 (15.2) | 12 (27.3) | 5 (19.2) |
| No change | 10 (11.0) | 4 (8.7) | 1 (2.3) | 1 (3.8) |
| Slightly better | 7 (7.7) | 0 (0) | 0 (0) | 0 (0) |
| Moderately better | 2 (2.2) | 2 (4.3) | 1 (2.3) | 1 (3.8) |
| Much better | 1 (1.1) | 0 (0) | 0 (0) | 0 (0) |
| Missing data | 1 (1.1) | 2 (4.3) | 2 (4.5) | 0 (0) |

**Supplementary items 5: Relative importance of pandemic factors to exacerbation of ED symptomatology**

This table includes only those participants who reported their ED symptomatology had worsened with the pandemic. Participants rated the importance of each aspect of the pandemic in relation to their ED, from 1 (not important at all) to 100 (very important). Average ratings for each factor are shown, along with SD in brackets, for all participants pooled and for each diagnostic group.

| Aspects of  the pandemic | All participants  (n = 172) | AN  (n = 70) | BN  (n = 38) | BED  (n = 40) | OSFED  (n = 24) |
| --- | --- | --- | --- | --- | --- |
| Disruption to living situation | 62.3 (27.6) | 61.3 (27.2) | 67.3 (27.3) | 61.5 (26.0) | 58.8 (32.3) |
| Change in social support | 55.9 (31.7) | 58.7 (30.6) | 57.5 (30.2) | 50.0 (34.7) | 54.7 (32.9) |
| Change in physical activity | 69.8 (30.1) | 68.4 (27.7) | 77.2 (26.5) | 64.7 (34.0) | 70.5 (34.7) |
| Change in food availability | 55.0 (34.5) | 51.0 (33.5) | 49.7 (33.5) | 68.1 (34.6) | 53.6 (35.8) |
| Exposure to triggering messages | 57.0 (34.9) | 64.7 (30.7) | 51.3 (37.7) | 45.0 (37.0) | 63.5 (33.0) |
| Disruption to routine | 71.2 (27.9) | 74.6 (22.9) | 70.4 (27.7) | 74.2 (27.4) | 57.8 (37.9) |
| Emotions | 71.8 (26.5) | 72.3 (28.6) | 69.9 (28.0) | 74.9 (22.1) | 68.3 (25.5) |
| Change in treatment | 41.3 (35.7) | 44.6 (36.9) | 41.1 (31.7) | 36.8 (38.2) | 39.1 (35.6) |
| Physical health concerns | 52.4 (34.8) | 55.3 (33.0) | 53.3 (38.0) | 52.8 (33.3) | 40.3 (37.5) |

**Supplementary items 6: Regression models split by diagnosis category**

**Model 1: DASS.** In participants with AN, we found that lack of access to emotion regulation strategies (B=3.08, *p<.*001), non-acceptance of emotions (B=1.9, *p=.*002), and lack of emotional clarity (B=2.6, *p<.*001) were significant predictors of poor mental health as measured by the DASS (*Adj. R^2^* = .520, *F*(4, 86)=25.4, *p<*.001). We found that lack of access to emotion regulation strategies was the only significant predictors of the DASS for both participants with BN (B=3.20, *p=.*002, *Adj. R^2^* = .495, *F*(2, 43)=16.8, *p<.*001) and BED (B=6.0, *p<.*001, *Adj. R^2^* = .496, *F*(1, 42)=43.3, *p<.*001). Finally, for participants with OSFED, non-acceptance of emotions (B=5.4, *p<.*001) was the only significant predictor of poor mental health during lock down (*Adj. R^2^* = .493, *F*(1, 24)=25.3, *p<.*001).

**Model 2: Self-reported change in symptoms.** Emotion regulation strategies were not found to be significant predictors of self-reported changes in eating disorder symptoms for our participants with BED (*Adj. R^2^* = .030, *F*(1, 40)=2.26, *p=*.140) and OSFED (*Adj. R^2^* = .089, *F*(1, 24)=3.4, *p=*.076). In participants with AN, lack of access to emotion regulation strategies (B=-0.23, *p=.*003) as well as difficulties maintaining goals when upset (B=0.20, *p=.*002) were small but significant predictors of self-reported changes in eating disorder symptoms (*Adj. R^2^* = .118, *F*(3, 86)=5.0, *p=*.003). In participants with BN, lack of access to emotion regulation strategies (B=0.13, *p=.*016) was the only significant predictors of self-reported changes in eating disorder symptoms (*Adj. R^2^* = .109, *F*(1, 42)=6.25, *p=*.016).

**Model 3: EDEQ.** We found that difficulties maintaining goals when upset was the only significant predictors of eating psychopathology in AN (B=0.14, *p<.*001; *Adj. R^2^* = .126, *F*(1, 89)=14.0, *p<*.001), BN (B=0.11, *p=.*023; *Adj. R^2^* = .092, *F*(1, 44)=5.58, *p=*.023), BED (B=0.16, *p=.*004; *Adj. R^2^* = .160, *F*(1, 42)=9.20, *p=*.004) and OSFED (B=0.22, *p<.*001; *Adj. R^2^* = .390, *F*(1, 24)=17.0, *p<*.001).
